# The effects of printing orientation on the electrochemical behaviour of 3D printed acrylonitrile butadiene styrene (ABS)/carbon black electrodes

**DOI:** 10.1038/s41598-018-27188-5

**Published:** 2018-06-14

**Authors:** Hairul Hisham Bin Hamzah, Oliver Keattch, Derek Covill, Bhavik Anil Patel

**Affiliations:** 1 School of Pharmacy and Biomolecular Sciences, University of Brighton, Brighton, East Sussex UK; 2 School of Computing, Engineering and Mathematics, University of Brighton, Brighton, East Sussex UK; 3 Centre for Stress and Age-Related Diseases, University of Brighton, Brighton, BN2 4GJ UK; 4 Present Address: School of Chemical Sciences, Universiti Sains Malaysia (USM), 11800 Pulau Pinang, Malaysia

## Abstract

Additive manufacturing also known as 3D printing is being utilised in electrochemistry to reproducibly develop complex geometries with conductive properties. In this study, we explored if the electrochemical behavior of 3D printed acrylonitrile butadiene styrene (ABS)/carbon black electrodes was influenced by printing direction. The electrodes were printed in both horizontal and vertical directions. The horizsontal direction resulted in a smooth surface (HPSS electrode) and a comparatively rougher surface (HPRS electrode) surface. Electrodes were characterized using cyclic voltammetry, electrochemical impedance spectroscopy and chronoamperometry. For various redox couples, the vertical printed (VP) electrode showed enhanced current response when compared the two electrode surfaces generated by horizontal print direction. No differences in the capacitive response was observed, indicating that the conductive surface area of all types of electrodes were identical. The VP electrode had reduced charge transfer resistance and uncompensated solution resistance when compared to the HPSS and HPRS electrodes. Overall, electrodes printed in a vertical direction provide enhanced electrochemical performance and our study indicates that print orientation is a key factor that can be used to enhance sensor performance.

## Introduction

Development of electrochemical sensors with complex geometries is challenging. This is most commonly approached by developing of conductive composite sensors that have the scope to be moulded into any shape and size. Composite electrodes are defined as a surface that consists of an ordered arrangement (array) or a random arrangement (ensemble) of conductor regions, typically micrometres in dimension, separated from one another by an insulator^1^. Early composite electrodes were carbon paste electrodes^2^, however over the years various conductive and insulative materials have been utilised^3–6^. Although these electrodes have been highly beneficial as they are easy to make in any geometry, and are mechanically robust, they often have high performance variability due to differences in the homogeneity of the material and electrode surface from batch to batch. Alongside this, they have increased resistance compared to solid conductive materials, therefore posing challenges to our current approaches for manufacturing composite eletctrodes^1^.

With improved availability of 3D printers and increased diversity of conductive filaments been made available, addictive manufacturing has become a viable cost-effective approach for design and development of electrochemical sensors of complex geometries with high reproducibility. There have been numerous studies that have utilised 3D printing for sensing, where carbon and metallic materials have been used for printing electrodes^7,8^. A polystyrene 3D-Printed electrochemical device with embedded carbon nanofiber-graphite-polystyrene composite conductor electrode, showed to have good signal to background voltammetric responses for detection of aqueous Pb^2+^ via anodic stripping^9^. 3D printed metal electrodes have been shown to be suitable for the measurement of various analytes, where improved analytical performance and electrode stability compared to glassy carbon electrodes were shown^10,11^.

These studies clearly highlight the performance benefits of 3D printed materials but did not indicate if the approach taken to create 3D printed geometries could have an influence on the electrochemical behaviour of the conductive material. One study has explored how the anisotropy in between layers of 3D printed acrylonitrile butadiene styrene (ABS)/carbon black composite structure would alter the resistance of the material. This study showed reduced resistivity in vertical printed direction than the horizontal direction^12^, however they did not explore the electrochemical performance.

Therefore, the aim of our study was to explore the electrochemical behaviour of 3D printed ABS/carbon black surfaces that were printed either horizontally or vertically. The electrodes were investigated using various redox couples with a range of electrochemical approaches to investigate their conductive behaviour and ability to serve as sensors for electrochemical analysis.

## Results and Discussion

### Investigation of the conductive properties and electrochemical behaviour of 3D printed electrodes

**Figure 1:**
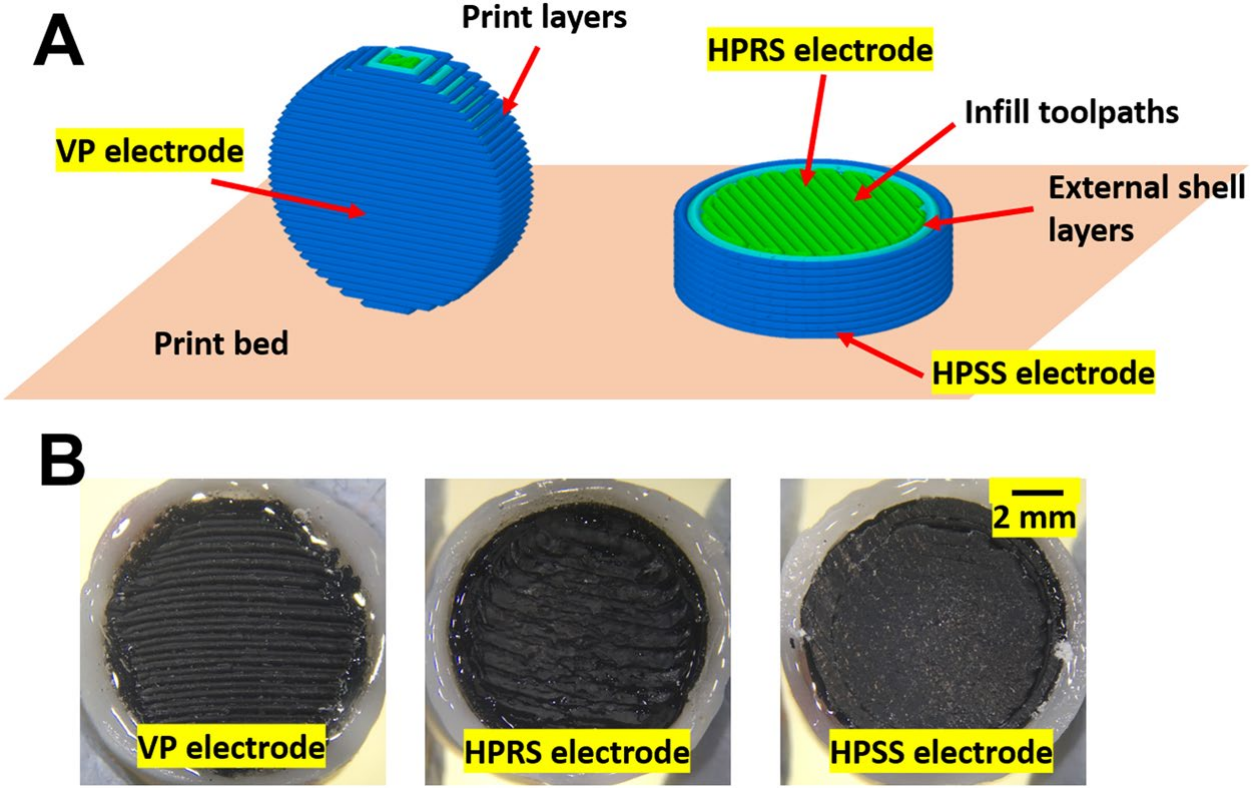
3D printed electrodes. (**A**) shows the approach in which the horizontal and vertical print of the ABS/carbon black material was used to generate vertical printed (VP), horizontal printed smooth surface (HPSS) and horizontal printed rough surface (HPRS) electrodes. The cross section of the electrode is shown on the right. (**B**) Photographs of 3D printed carbon black/ABS electrodes showing electrodes printed vertically and horizontally.

Figure 1 shows the various 3D printed orientations resulting in the production of the vertically printed (VP) electrodes and the horizontally printed electrodes, which generated two different surfaces for electrochemical characterisation. With horizontal direction of printing, we obtained a horizontal printed smooth surface (HPSS) and horizontal printed rough surface (HPRS) electrode. The smooth surface of the HPSS electrode was generated due to the first layer of the fused deposition modelling (FDM) print being thinner than the main layer thickness. In this study the initial layer was set to 90% (so on a 0.3 mm layer thickness print, the first layer is approximately 0.27 mm) but the same volume of material is extruded. This deliberate over-extrusion results in the material pressing firmly against the print bed, to bind it in place during printing. As print beds are usually glass, this also results in a very smooth lower surface on the HPSS electrode, as the material is being pressed against the bed. By comparison, the top surface is not subjected to this, resulting in the infill paths being more clearly defined, creating a rougher surface texture of the HPRS electrode.

**Figure 2:**
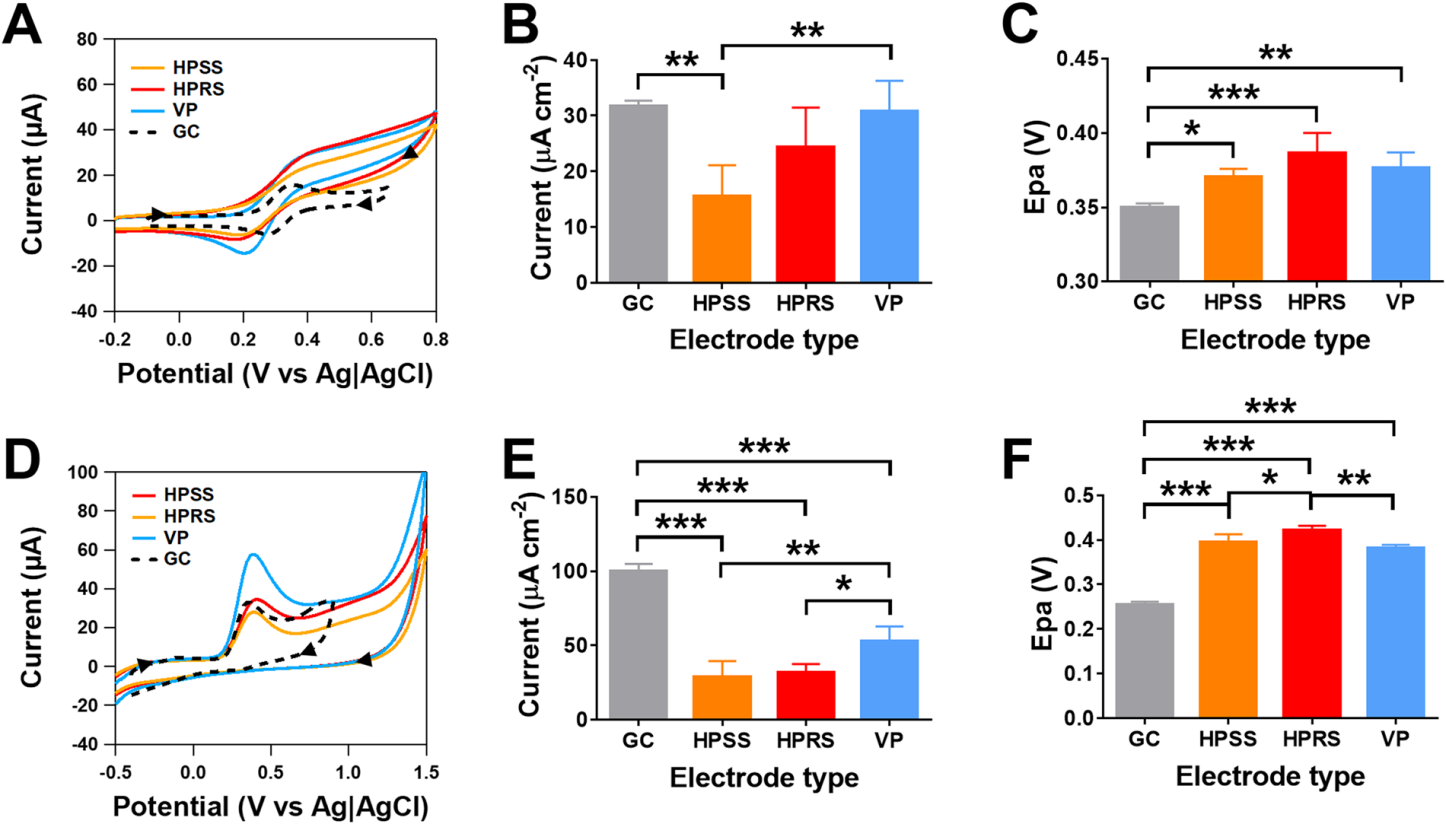
Cyclic voltammetric responses on the printed electrodes. Voltammograms of glassy carbon (GC), vertical printed (VP), horizontal printed rough surface (HPRS) and horizontal printed smooth surface (HPSS) electrodes for (**A**) 1 mM ferrocene carboxylic acid in 0.1 M NaOH and (**D**) 1 mM serotonin hydrochloric acid in tris buffered saline (0.05 M tris and 0.15 M NaCl), measured at a scan rate of 100 mV/s. Responses of (**B**) anodic peak current normalised to electrode surface area (*i*_pa_) and (**C**) anodic peak potential (*E*_pa_) for 1 mM ferrocene carboxylic acid. (**E**) anodic peak current normalised to electrode surface area (*i*_pa_) and (**F**) anodic peak potential (*E*_pa_) for 1 mM serotonin hydrochloric acid. Statistical analyses were performed using one-way ANOVA followed by a post hoc Tukey test. Data are shown as mean ± S.D., n = 4, *P < 0.05, **P < 0.01 and ***P < 0.001.

The electrochemical filament which is utilised to print the electrodes and serves as the raw (control) material was accessed to compare with the printed electrodes. Figure S1 shows electrochemical response of ferrocene carboxylic acid on the ABS/carbon black filament utilised for printing. The response is highly resistive with no clear presence of oxidation or reduction peaks over the wide potential window utilised. This suggest that the base conductive material may have a low percentage of carbon black and/or the composite material does not have sufficient conductive pathways to meet the percolation threshold to show conductive behaviour as other studies have demonstrated using graphite powder based composite electrodes^13,14^. The 3D printed electrodes were assessed using surface-insensitive ferrocene carboxylic acid (Fig. 2A) and serotonin hydrochloric acid (Fig. 2D), which requires a surface specific interaction based on previously published studies to characterise solid and composite carbon-based materials^6,15,16^. In both compounds, faradaic responses were observed suggested that there is a sufficient percentage of carbon black, but the extruded material doesn’t provide sufficient conductive pathways throughout the material.

The background potential window for the different printed orientation were similar, with the potential window being between −1.0 and +1.2 V (Figure S2), which is similar for most sp^2^ carbon materials. Figure 2B shows the anodic peak current normalised for electrode surface area (*i*_*pa*_) for ferrocene carboxylic acid, where there was a significant increase in the *i*_pa_ for the VP electrode when compared to HPSS electrode (p < 0.01, n = 4). There was also a significant decrease in the current observed on the HPSS electrode when compared to a glass carbon (GC) electrode (p < 0.01, n = 4). A significant increase in the anodic peak potential (*E*_pa_) was observed between all printed electrodes and GC electrode (p < 0.001, n = 4), however no significant differences in the *E*_pa_ of ferrocene carboxylic acid were observed between the VP, HPSS and HPRS electrodes (Fig. 2C). There was a significant decrease in the ratio of the anodic and cathodic peak current (*i*_pa_/*i*_pc_) for HPRS electrode when compared to the GC (p < 0.001), HPSS (p < 0.05) and VP electrodes (p < 0.01, n = 4, Figure S3A). No significant differences in the difference between the anodic and cathodic peak potential (Δ*E*_p_) were observed between the three printed electrodes, however the Δ*E*_p_ was significantly larger than the GC electrode (p < 0.001, n = 4, Figure S3B).

Serotonin undergoes an electrochemical chemical reaction resulting in an irreversible redox response following adsorption on the electrode surface^17,18^. The *i*_pa_ of serotonin on the GC electrode was significant higher than all printed electrodes (p < 0.001, n = 4). The VP electrodes had a significantly higher *i*_pa_ than HPRS (p < 0.05) and HPSS electrodes (*p < *0.01, n = 4, Fig. 2E). The *E*_pa_ of serotonin was significantly higher in all three printed electrode surfaces when compared to the GC electrode (p < 0.001, n = 4). The HPRS electrode had a significantly higher *E*_pa_ when compared to HPSS (p < 0.05, n = 4) and VP electrodes (p < 0.01, n = 4, Fig. 2F).

Our findings suggest that for either a surface insensitive or sensitive redox couple, the VP electrode provides the greatest current response when compared to the horizontally printed electrodes. When compared to the GC electrode, the current observed when normalised to the electrochemical surface area, showed identical responses for VP electrodes in the case of ferrocene carboxylic acid, but reduced response in the case of serotonin which relies on adsorption. This may be due to the enhanced surface area of the vertical electrodes due to the groves between each of the print layers or due to printing orientation providing enhanced conductive pathways when compared to the horizontal printed materials. There were no differences in the current between the HPSS and HPRS electrodes, suggestive a rougher (and potentially higher) surface area was not a key factor in enhancing the current response and the properties of the entire material are as essential to conductive performance. Limited differences in the oxidation peak potentials were observed on all three electrode surfaces that were generated by 3D printing. However, printed electrodes had much larger oxidation peak potentials (for both serotonin and ferrocene carboxylic acid) than that of the GC electrode. This suggest the 3D printed materials are kinetically limited in comparison to the GC electrode.

### Understanding the changes in double layer capacitance (Cdl) between the 3D printed electrodes

Since there was significant difference in the voltammetric behaviour between the VP, HPRS and HPSS electrode, we investigated if these changes were due to enhanced electroactive surface area, as the roughness between the three electrode is varied.

**Figure 3:**
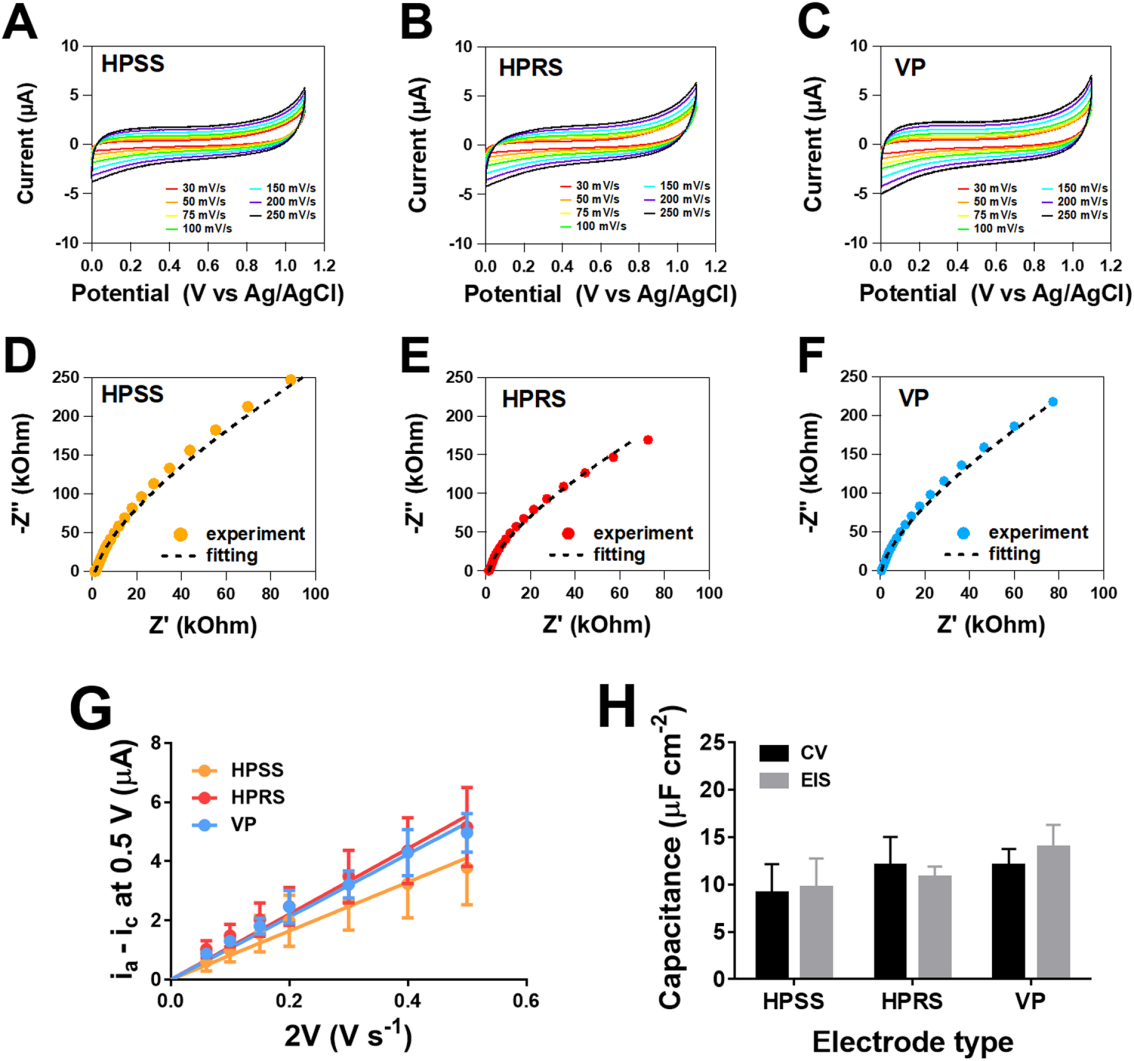
Determination of the double layer capacitance (*C*_dl_) between the printed electrodes. (**A–C**) shows cyclic voltammograms at 30 mV/s to 250 mV/s and (**D–F**) shows Nyquist plots for the VP, HPRS and HPSS electrodes. CVs were measured in 1 M KCl from 0 to 1.1 V vs Ag/AgCl. The EIS measurements were made on a frequency range from 100 kHz to 0.1 Hz, using a modulation amplitude of 5 mV. (**G**) shows plots of Δi vs 2 V for VP, HPRS and HPSS electrodes in order to determine the *C*_dl_. (**H**) shows comparisons of the *C*_dl_ when normalised to the electrode surface area of the three-printed electrodes. Statistical analyses were performed using two-way ANOVA. Data shown as mean ± S.D., n = 4.

As capacitance is directly proportional to the electroactive area at the electrode surface^19^, we measured *C*_dl_ by cyclic voltammetry (CV) and electrochemical impedance sprectroscopy (EIS) techniques as shown in Fig. 3. In CV measurements, the *C*_dl_ was obtained from measurement of the anodic and cathodic currents on the VP, HPRS, HPSS electrodes at varying scan rates in 1 M KCl, which served as an electrolyte solution (Fig. 3A–C). Figure 3G shows the plot of difference in anodic and cathodic current (Δi) at 0.5 V versus the scan rate (2 V), where a linear relationship was observed for each printed electrode. These responses were verified by obtaining *C*_dl_ using EIS. Figure 3D–F shows the Nyquist plots for VP, HPRS and HPSS electrodes where good agreement between the experiment responses and fitting data using a *RC* equivalent electric circuit (Figure S4) were observed.

The *C*_dl_ normalised to the electrode surface area for the three printed electrodes by CV and EIS is shown in Fig. 3H. There was no significant difference between the two approaches and no difference in the *C*_dl_ between the VP, HPRS and HPSS were observed. These findings indicate that the active electrochemical surface on the three printed electrodes are identical and therefore the electrode roughness and variations in the surface area of the electrodes are not reflective of the electrochemical surface area. This would indicate that the differences observed voltammetric behavior between the printed electrode is linked to the homogeneity and/or conductive pathlength within the composite material and how this may be altered during the printing process.

### Determination of the solution and charge transfer resistance between the 3D printed electrodes

**Figure 4:**
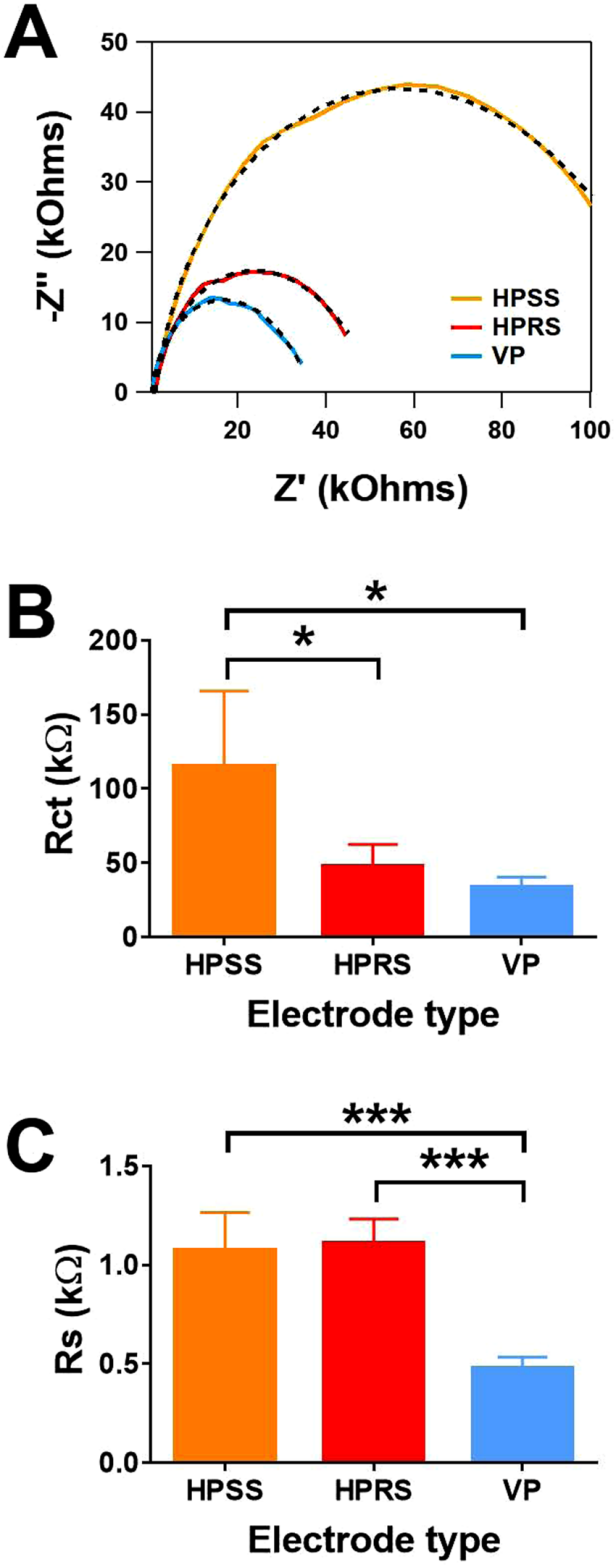
Determination of charge transfer and solution resistance. (**A**) Nyquist representations of the impedance spectra at ~0.6 V *vs* Ag|AgCl in 10 mM of K_4_[Fe(CN)_6_]/K_3_[Fe(CN)_6_] in 1 M KCl for VP, HPRS and HPSS electrodes. The measurements were made at a frequency range from 100 kHz to 0.1 Hz with a modulation amplitude of 5 mV. (**B**) shows a comparison of the charge transfer resistance (*R*ct), determined by EIS fitting analysis and (**C**) is a comparison of the uncompensated solution resistance (*R*s), also determined from EIS fitting analysis. Statistical analyses were performed using one-way ANOVA followed by a Tukey test. Data are shown as mean ± S.D., n = 4, *P < 0.05 and ***P < 0.001.

Figure 4A shows Nyquist plots for the VP, HPRS and HPSS electrodes, from which the uncompensated solution resistance (*R*_s_) and interfacial charge-transfer resistance (*R*_ct_) was obtained. Ideally, a typical Nyquist plot should consist of a semicircle plot at high frequencies and the Warburg line. The Warburg line describes the semi-infinite diffusion of the redox solutes from the bulk solution to the electrode at low frequencies and is characterised by a 45° linear region in the Nyquist plot. In contrast, for our 3D printed electrodes, a semicircle was obtained, and the Warburg line was completely absent. As the Warburg line didn’t appear from the impedance signals, it can be concluded that electrochemical reactions occurring on the VP, HPRS and HPSS were controlled by charge transfer, not by the mass transfer^20^.

The semicircles in Fig. 4A clearly indicated that there were significant differences of Z’ between the three printed electrodes. The shifting of Z’ indicates that the charge transfer resistance (*R*_ct_) has considerably increased. To obtain the electrochemical parameters from the Nyquist plots such as *R*_s_ and *R*_ct_, we fitted the experiment data using a general Randles circuit of which the circuit is shown in Figure S5. The *R*_ct_ was signficantly higher for HPSS electrodes when compared to HPRS and VP electrodes (p < 0.05, n = 4, Fig. 4B). *R*_*s*_ was signficantly lower for the VP electrode when compared to the HPSS and HPRS electrodes (p < 0.001, n = 4, Fig. 4C).

Our findings clearly indicate that the VP electrode has the lowest *R*_ct_, when compared to the two horizontal printed electrodes, which indicates that the VP electrode is a more favourable for electron transfer. This is most likely due enhanced homogenatity of the conductive parts within the non-condutive parts of the material generating more conductive pathways as a nature of the vertical printing. The reduced *R*_s_ may be a nature of the groves present on the surface due to vertical printing, which may support the genration of the electrified interface. A high degree of variability in the *R*_ct_ in the HPSS may be due to the heterogeneous nature of the electrode surface, which can potentially hinder electron transfer. Our findings are supportive of a study that explored the anisotropy between layers of 3D printed ABS/carbon black composite structure, where they also observed that reduced resistivity in vertical direction of single print layers than the horizontal direction^12^.

### Determination of the *RC* time constant on the 3D printed electrodes

**Figure 5:**
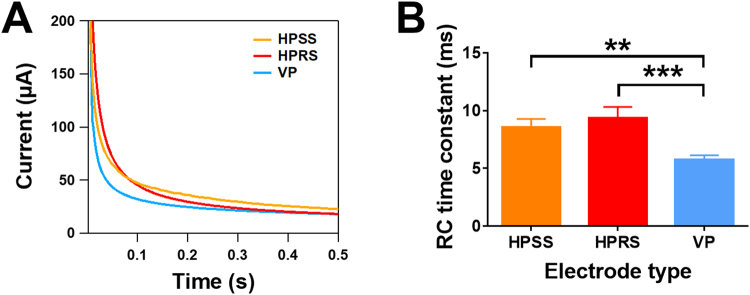
Chronoamperograms for V, HR and HS (**A**) on the oxidation of 1 mM ferrocyanide in 1 M KCl by stepping potential from 0 to 1.2 V vs Ag/AgCl for 1 s. (**B**) shows a comparison of the *RC* time constant, calculated from plots of *ln*i vs *t*. Statistical analysis was performed using one-way ANOVA followed by a Tukey test. Data are shown as mean ± S.D., n = 4, *P < 0.05 vs vertical surface.

The *RC* time constant was measured using chronoamperometry. Figure 5A shows chronoamperograms of 1 mM ferrocyanide from VP, HPRS and HPSS electrodes, where two types of current decay, corresponding to double-layer charging and Faradaic current flow are present. Both currents are separated by a transient time which was monitored. There were significant differences in the RC transient time of HPRS (p < 0.001) and HPSS (p < 0.01) electrodes when compared to VP electrodes (n = 4, Fig. 5B). In HPRS and HPSS electrodes, the capacitive currents took longer to fall off and stabilise, which compliments the larger *R*_s_ obtained for these electrodes when compared to VP electrodes. Again, this data indicates the differences in the electrodes materials achieved through the printing orientation.

## Conclusion

Overall, our findings indicate that 3D printed conductive materials can produce useful sensors for electroanalytical purposes, and that print orientation can significantly influence the electrochemical behaviour. Printing of conductive carbon composite filaments into 3D structures enhanced the electrochemical behaviour of the base material. VP conductive carbon composite electrodes provided enhanced electrochemical performance when compared to horizontally printed electrodes. This VP electrode showed enhanced oxidation current for surface insensitive and surface dependant redox species, of which it was comparable to a glassy carbon electrode for the surface insensitive compound. No differences in the capacitance was observed between the printed electrodes, which was suggestive that no differences in the electrochemical active surface were observed even though there were distinct differences in the roughness of the electrode surfaces. The enhanced performance of the VP electrodes is most likely due to the internal structure of the material as the print layers were orientated in the direction that follows the conductive pathways from electrical connection to solution interface. Overall our findings indicate that printing direction can provide distinct differences in conductive behaviour which should be considered in the development of 3D printed electroanalytical sensors.

## Methods

### Fabrication of 3D printed carbon black/ABS electrodes

The electrodes were printed on a Wanhao Duplicator 4 with a 0.4 mm nozzle at 220 °C and a bed temperature of 50 °C. The layer height was set at 0.3 mm, with 2 shells (outer perimeter toolpaths) and 100% infill density. Two skirt outlines were used to prime the extruder. To compensate for the filler content of the material, retractions were disabled, and the extrusion multiplier was set to 1.1. This forces the printer to extrude 10% more material than necessary, which keeps the filament flowing more rapidly through the nozzle, reducing the chance of the filler material burning and jamming the machine. Electrodes were printed in either horizontal or vertical direction as shown in Fig. 1.

To print the vertical discs, a 5 mm flat was cut into the edge of the disc. This provided a sufficiently large surface area to bind the print to the print bed for the duration of printing. Printing a true circle was not possible, as the contact area between the disc and the bed would be too small to achieve adhesion.

ABS/Carbon Black filament was printed into discs of 10 mm diameter and 3 mm thickness. These discs were placed in acrylic tube and sealed with Araldite. Connection was achieved by attaching a copper wire to the electrode using CircuitWorks Conductive (Silver) epoxy.

### Electrochemical characterisation of 3D printed electrodes

Electrochemical assessments on the 3D printed electrodes were carried out using a three-electrode system, which consisted of a Ag|AgCl (3 M KCl) reference electrode, a platinum wire auxiliary electrode and the 3D printed electrodes (10 mm diameter and 3 mm thickness with a surface area of 78.5 mm^2^) as the working electrode. All the electrochemical experiments were carried out using a CH instrument potentialstat/galvanostat CHI 760E.

The electrochemical characteristics of the 3D electrodes were assessed using the well-understood redox couples of 1 mM ferrocene carboxylic acid in 0.1 M sodium hydroxide and 1 mM serotonin in Krebs’ buffer solution, pH 7.4 (117 mM NaCl, 4.7 mM KCl, 2.5 mM CaCl_2_, 1.2 mM MgCl_2_, 1.2 mM NaH_2_PO_4_, 25 mM NaHCO_3_ and 11 mM glucose). Cyclic voltammograms were performed over potential windows of −0.2 to 0.9 V for ferrocene carboxylic acid and −1 to 1.6 V for serotonin with a scan rate of 100 mV/s.

EIS measurements were performed in a mix of 0.5 mM potassium ferricyanide and 0.5 mM potassium ferrocyanide in 1 M KCl at a potential equal to the anodic potential of ~0.58 V. A range frequency of 100 kHz to 0.01 Hz and an amplitude of 5 mV were utilised.

The capacitance of the double layer (*C*_dl_) measurements were carried out by CV and EIS. The CV measurements were performed over a potential window of 0 to 1.1 V in 1 M KCl solution by varying the scan rates from 0.03 to 2.5 V/s. In contrast, EIS was carried out at a potential of 0.6 V in 1 M KCl solution. A range frequency of 100 kHz to 0.01 Hz and an amplitude of 5 mV were utilised.

### Data analysis

From the cyclic voltammetry measurements, the *E*_pa_, *E*_pc_, *i*_pa_ and *i*_pc_ was obtained from the voltammograms using CH1 760E software.

For measurement of the double layer capacitance, the difference between the anodic (I_a_) and cathodic (I_c_) currents at 0.5 V was determined from voltammograms obtained in 1 M KCl. This difference was divided by the scan rate (2 V) as shown in equation 1. Such measurements were obtained from voltammograms obtained from 30 to 250 mV/s. A plot of Δ*i* vs 2 V was obtained from which the slope was utilised to obtain the double layer capacitance.

1 $${C}_{dl}=\frac{{i}_{a}-{i}_{c}(\Delta i)}{2V}$$

For obtaining the values for the double layer capacitance, solution resistance and charge transfer resistance, the equivalent electric circuit employed in electrochemical impedance spectroscopy fitting analysis was stimulated within CH1 760E software (Figures S4 and S5).

By following the potential step in chronoamperometry, the charging current decays exponentially in time at a rate governed by *RC*, as expressed in Equation 2^21^.

2 $${i}_{c}=\frac{\Delta E}{{R}_{t}}{ex}{{p}}^{\frac{-t}{RC}}$$

where Δ*E* is the pulse amplitude, *R*_s_ is the uncompensated solution resistance and *C*_dl_ is double layer capacitance. Based upon Equation 2 the *RC* time constant for each electrode was determined by making a plot of ln*i* vs *t*^21^. Thus, from plots of ln*i* vs *t*, a linear regression line in the capacitive region was obtained. As indicated by Equation 2, a straight-line equation can be expressed as ln*i*_c_ = (−1/ *RC*) t + 1n(Δ*E*/*R*), where the gradient is 1/ *RC*.

For all measurements, the data was presented as mean and its associated standard deviation and statistically compared using one-way ANOVA test with post hoc Tukey tests where appropriate.

## Electronic supplementary material

Supplementary Information
