# Supplementary material for: The effects of printing orientation on the electrochemical behaviour of 3D printed acrylonitrile butadiene styrene (ABS)/carbon black electrodes
Source: Sci Rep. 2018 Jun 14;8:9135. doi: 10.1038/s41598-018-27188-5 (PMC6002470; doi:10.1038/s41598-018-27188-5)
Supplement: Supplementary file 1 — Supplementary Information [file 41598_2018_27188_MOESM1_ESM.docx]

**SUPPLEMENTARY INFORMATION**

**The effects of printing orientation on the electrochemical behaviour of 3D printed acrylonitrile butadiene styrene (ABS) / carbon black electrodes**

Hairul Hisham Bin Hamzah^1†^, Oliver Keattch^2†^, Derek Covill^2^ & Bhavik Anil Patel^1,*^

^1^School of Pharmacy and Biomolecular Sciences and

^2^School of Computing, Engineering and Mathematics, University of Brighton, Brighton, East Sussex, UK

**^†^ Joint first authors**

*** Correspondence to:** [b.a.patel@brighton.ac.uk](mailto:b.a.patel@brighton.ac.uk)

**
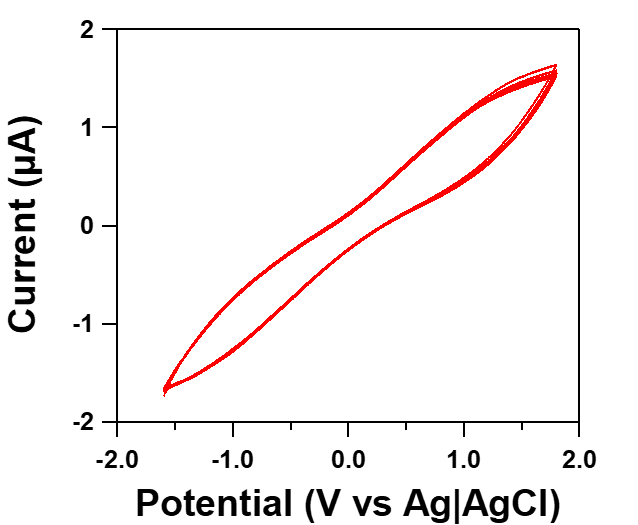
**

**Figure S1.** Voltammograms for acrylonitrile butadiene styrene (ABS) / carbon black print filament material utilised for development of 3D printed electrodes. Responses are shown in 1 mM ferrocene carboxylic acid in 0.1 M sodium hydroxide at a scan rate of 100 mV/s. Electrode diameter and thickness is 3 mm.

**
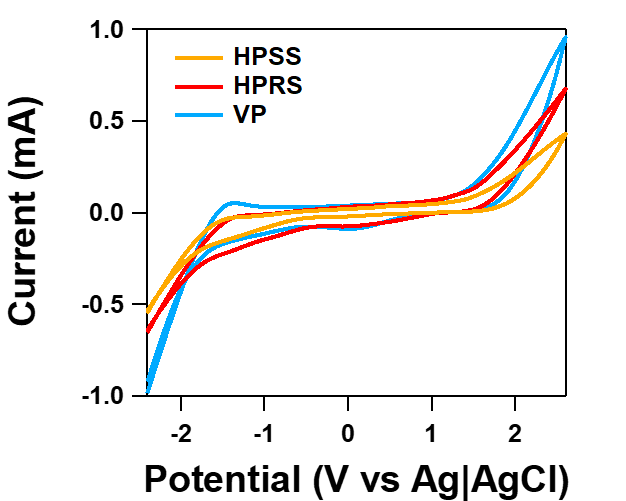
**

**Figure S2.** Voltammograms for vertical, horizontal smooth and horizontal rough 3D printed electrodes in 1 M KCl, showcasing the background potential window, which is similar for all three electrodes. Experiments carried out at scan rates of 100 mV/s

**
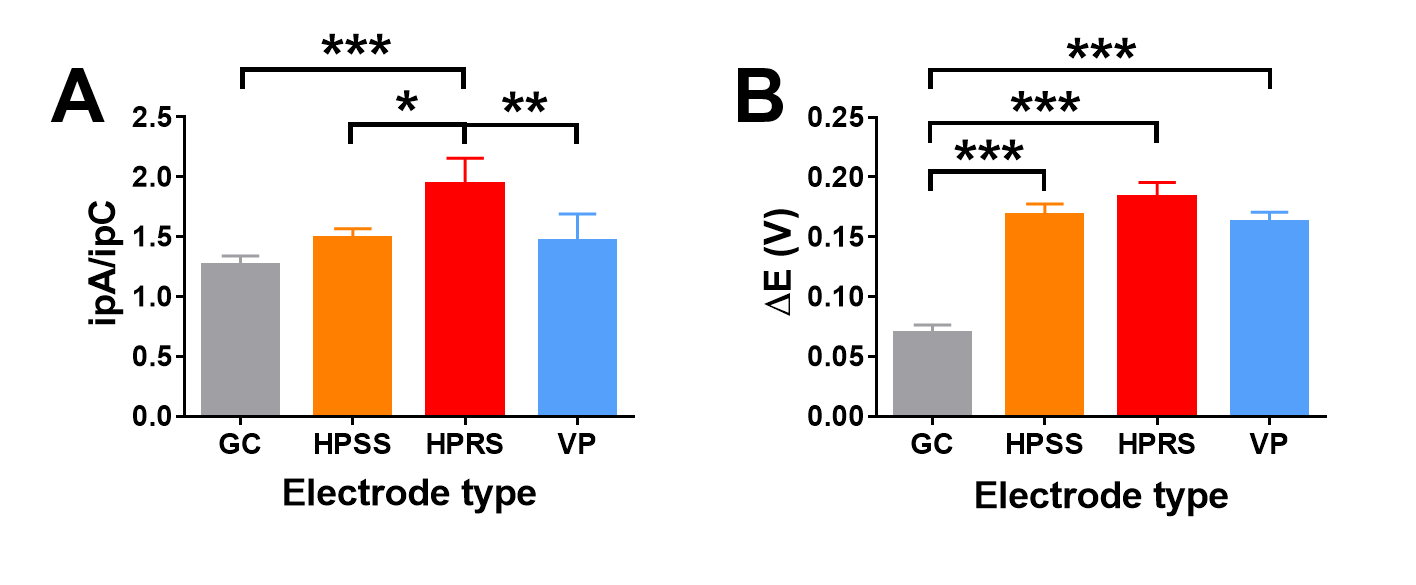
**

**Figure S3. Additional responses of performance parameters on 1 mM ferrocene carboxylic acid. (A)** Comparison of the ratio of *i*_pa_/*i*_pc_ on gassy carbon (GG), vertical printed (VP) electrode, horizontal printed smooth surface (HPSS) and horizontal printed rough surface (HPRS). (B) shows a comparison of the anodic and cathodic peak separation (Δ*E*_p_). Statistical analyses were performed using one-way ANOVA followed by a post-hoc Tukey test. Data shown as mean ± S.D., n = 4, and **P* < 0.05, ***P* < 0.01 and ****P* < 0.001.

**
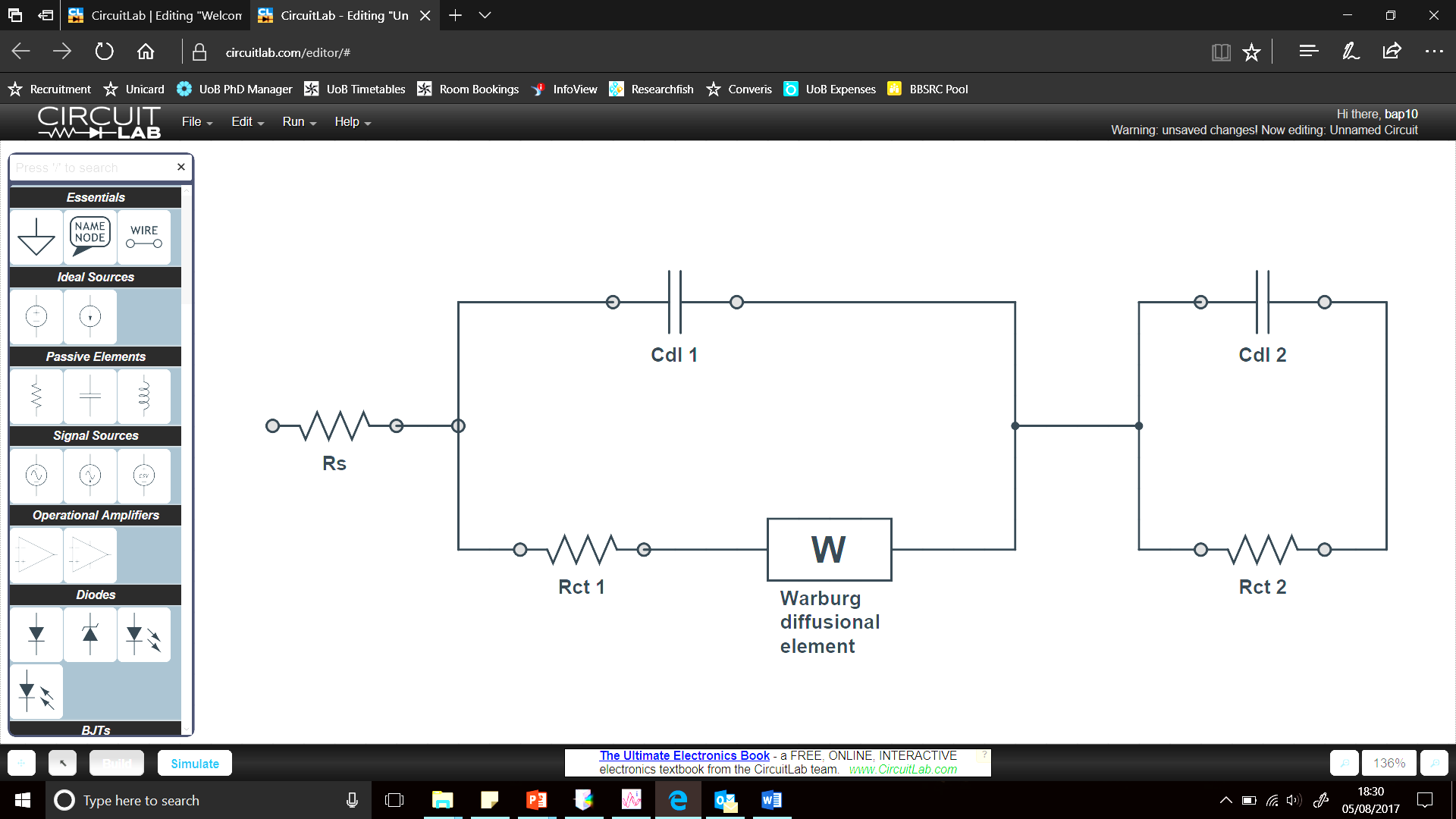
**

**Figure S4.** The best equivalent circuit used to fit the experimental data shown in Figures 3D-3F. *R*_s_ is the uncompensated solution resistance, *C*_dl_ 1 and 2 are the double layer capacitances, *R*_ct_ 1 and *R*_ct_ 2 are the charge transfer resistances and W is the Warburg diffusional element. W was used in the circuit in order the obtain the best-fitted data with the smallest error of fitting. The presence of W in the circuit may correspond to the diffusion process of protons and electrolyte ions to the electrode surface from the 1 M KCl solution in the diffuse layer.


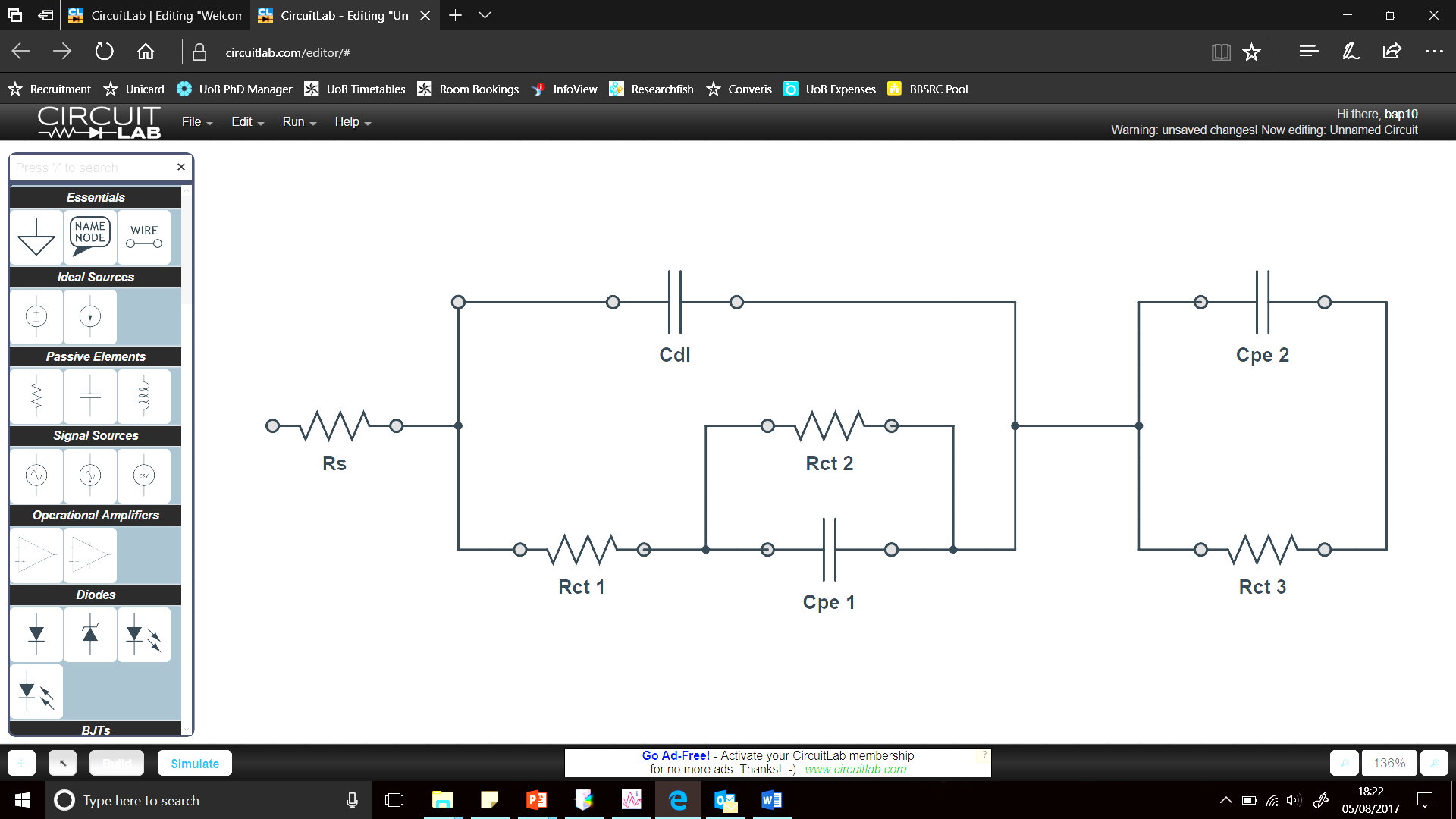


**Figure S5.** The best equivalent circuit used to fit the data shown in Figure 4a to the Faradaic impedance measurements in 10 mM of ferricyanide/ ferrocyanide solution (black lines). *R*_s_ is the uncompensated solution resistances, *C*_dl_ is the double layer capacitances, *R*_ct_ 1, *R*_ct_ 2 and *R*_ct_ 3 are the charge transfer resistances. CPE 1 and 2 are the constant phase elements.
